# Talks about Food

**Published:** 1892-03-26

**Authors:** 


### Talks about Food.

### V.—THE STAFF OF LIFE.

Every educated person nowadays knows, or ought to know, that to speak of our present-day bread as the "staff of life" is to use a bit of a misnomer. The world has been taught, and very rightly too, to look upon wheat as almost as much a typical food as is milk, or an egg; and a proof of how deeply this teaching has sunk into men's minds may be found in the German legends of the miserable fate which befell "The Girl who trod on Bread," evidently a quite unnatural outrage, in the minds of old-world men—on a level, say, with "seething a kid in his mother's milk."

But then the loaf which this unnatural girl trod under foot was, we may confidently assert, a brown loaf—not the white bread that is now eaten in almost every English household, rich or poor, which has been carefully deprived by the miller of some of its most valuable properties, being thus transformed, as has been well said, from a "staff of life" into a "broken reed."

Not that this is of any very great importance to the well-to-do classes; and herein, perhaps, lies part of the difficulty of remedying the mischief. Those who live upon a mixed and generous diet can afford to waste such portions of wheat as are not palatable to them; and indeed they cannot be said to be guilty of waste at all, for the rejected bran and pollard are used for feeding other animals, which, in their turn, supply to their masters the elements that are lacking, so that it may very well be argued that a man who eats white bread, and feeds his poultry on pollard, really eats that pollard himself, merely putting it through a preliminary process, pleasing both to himself and to the chickens.

But where the really poor are concerned, the case is very different. When wages go down, or employment fails, other articles of diet are docked off one by one, until only bread remains; and, indeed, there are large numbers of persons in England who, as a permanent thing, can get but very little else. To them, the comfortable reflection that the food rejected by the baker is used up in another way is no more helpful than were the explanatory statements made by the impossibly atrocious Alderman in May Kendall's "In a Garret," who pointed out that, seeing that seven hours' sleep per night was highly conducive to the welfare of the human race, and seeing also that it was impossible for the conductors of his trams to take more than three, he did his level best to redress the balance by taking eleven himself! Not that the working man sees the injury which this craze for white bread is doing him. He does not know—how should he?—that the outer portion of the grain of wheat is rich in flesh and bone forming substance, and that by insisting upon its removal, he is not only depriving himself of nutriment, but also laying up trouble for himself with regard to his children, who can no more make strong and healthy bones without the wherewithal, than can a hen lay hard-shelled eggs without a due supply of lime or chalk. The question is one having an important bearing, then, upon the general health of the people. Diseases of the bones, rickets, and defective teeth seem to become more common year by year; and when we are told by doctors that a better kind of bread would "prevent disease and deformity," and that "where animal food constitutes either no part or a very small part of the diet, the importance of the nitrogenous and mineral components of the outer layers of the corn-grains is great," we must surely see that there is work here which urgently needs to be done.

On economical grounds, also, a change from white to brown or whole-meal bread would be of great value, not only because anything that tends to make our working classes stronger increases their capacity for work, but because a really satisfying diet can be obtained at a much lower cost where whole-meal bread is used, owing to its not needing to be supplemented, as white bread must be, with a large amount of other food.

It ought not to be so very difficult to induce our working classes to make some change in this respect, for the habit of eating white bread is not of very long standing after all. In the olden days, the principal food of the poor was brown bread; and in "Wilks' Encyclopædia," published in 1810, "household bread" is described as made from the whole of the grain, while even "wheaten bread" had only the coarse bran removed from it. We remember reading, several years ago, an interesting bit of autobiography, taken down from the lips of a labourer some eighty years of age, who graphically described the condition of the poor in his young days. "In 1812," he said, "wages were so low, bread so dear, and my father's family so large, that I had to turn out to work, though only seven years of age. I earned one-and-sixpence a week, and when I brought home my wages on Saturday night, I was sent to buy a four-pound loaf, for which I paid one-and-fivepence."

It seems almost incredible to us now that the labourers could have lived at all, with low wages, and bread at such a price; but, doubtless, the fact that this dear bread was of a more nutritive quality than our cheap white loaf was really of great service to them, little though they may have dreamed of it.

It is a very significant fact, and one well worth impressing on the minds of the people, that, as Magendie has proved, a dog fed on white bread alone is simply starved to death, while his health does not suffer at all if his food consists of bread made of unbolted flour. Another interesting fact is told us by a French physician, viz., that during the Crimean War, the Russian prisoners, who had been accustomed to live on coarse brown bread, could not get on at all on rations of white bread, such as the French received, and had to be given an increased quantity.

It is well worth our while, then, to do all we can to bring English people back to more sensible habits. Some persons find that they cannot eat real whole-meal bread on account of the irritation caused by the outside covering of all, which is of a flinty nature, and contains little or no nutriment; but this can easily be removed by the process known as decortication, and the wheat-meal bread recommended by the Bread and Food Reform League is a really excellent food in all respects.

This has been a hard winter—a winter in which wheat has been less plentiful, and therefore dearer. Such reasons afford excellent opportunities for trying what we can do in persuading people to get the full value out of this wheat and to make it go as far as it will, which is a very long way further than at present most of them have any idea of.

